# Collecting behavioral evidence from a highly mobile and seasonal population: A protocol for a survey on quad bike injuries

**DOI:** 10.1371/journal.pone.0298059

**Published:** 2024-03-04

**Authors:** Preetha Menon, Mohamed El-Sadig, Marwan F. Albastaki, Humaid Alzaabi, Saleh Alhammadi, Mansour Almehrzi, Hamed Aljanaahi, Rami H. Al-Rifai, Emad M. Masuadi, Michal Grivna

**Affiliations:** 1 Institute of Public Health, College of Medicine and Health Sciences, United Arab Emirates University, Al Ain, United Arab Emirates; 2 College of Medicine and Health Sciences, United Arab Emirates University, Al Ain, United Arab Emirates; Sunway University, MALAYSIA

## Abstract

**Background:**

Quad bikes are popular recreational, four-wheeled bikes in the Middle East. Injury prevention programs targeting quad bike crashes in the United Arab Emirates (UAE) need evidence about the risk factors and behaviours associated with these crashes in the target population. This is a protocol for a study aiming to investigate quad bike rider behaviours and to assess the risk factors associated with related injuries in the UAE.

**Methods:**

This is a cross-sectional observational study aiming to describe a seasonal sport in a desert environment. With an estimated sample size of 451, the survey will follow a three-stage, location-based sampling strategy using the line-transect method. A sampling frame of desert locations with high injury incidences was developed, using Dubai ambulance injury records. Further expansion of the sampling frame was participatory, involving police, enthusiasts, emergency responders and gas station employees. The data collection will be limited to the winter months in fifteen high-injury desert locations across three major Emirates in the UAE. Trained researchers will observe the riders directly in the desert to note their riding habits, followed by a researcher-administered interview on riding and injury history. The interviews will be administered in Arabic and English using Qualtrics software on handheld tablets with offline and online entry mode. In addition, paper-based entry with the same format will be used as a contingency in busy quad bike locations.

**Conclusion:**

The objective of this study protocol is to develop a comprehensive survey that will furnish substantial evidence for the formulation of effective injury prevention strategies. To enhance the credibility of the recorded riding behaviors, field observations will be employed. The uniqueness of this study lies in its innovative sampling strategy, custom-tailored to accommodate the highly mobile and transient population of desert bikers in the UAE.

## 1. Introduction

Quad bikes are straddle-seated vehicles designed for use on uneven terrain. Though envisioned for the agricultural sector, it has become a popular recreational sport in many regions of the world [1], especially the Middle East. The growing popularity of quad bike driving is seen in older age groups and young adolescents [2]. With its desert ecosystem and increasing recreational use, intervention strategies in the Middle East require a closer look at the unique risk factors relating to quad bike crashes. A systematic review of quad bike injuries revealed an alarming absence of research on quad bike crashes in the region [3]. Out of 196 studies exploring the risk factors of quad bike crashes across the globe, only one study briefly analysed the magnitude and risk factors of quad bike crashes in the Middle East was cited [4]. Evidence on quad bike crashes, their risk factors, societal burden and the most effective measures to control the problem in the UAE is minuscule. This research attempts to fill the gap in the existing literature on quadbikes injuries in the UAE.

However, several potential challenges exist, especially the most effective sampling method from such a highly mobile and dispersed population in the desert. For example, quad bike riders may have a regular riding neighbourhood near their home or may prefer to try out the more challenging dunes away from home. As a consequence, riding locations may vary, making neighbourhood-based street intercept and area sampling methods unreliable [5–7]. Likewise, the quota sampling methods may not be suitable for a population where quad biking is limited to a specific demographic group and not practised in all households. Thus, implementing traditional probability sampling methods using population registries or neighbourhood household surveys may compromise the representativeness of the resulting sample. Quad bike riders are also known to be heterogeneous in a country like the UAE, with a predominant proportion of tourists and expatriate populations. The absence of a fixed sampling frame for quad bike riders makes traditional sampling using a population register redundant [6, 8, 9]. Finally, similar to other outdoor sports in the Middle East, quad biking is a seasonal activity enjoyed during the cooler winter months. The riding season starts in October and ends in April, each year. As a result of the seasonality of this recreational sport, riders might be owners or hirers of quadbikes for a day during the weekend. Thus, quad bikes riding could be considered as infrequent and irregular activity. Comparatively, the sampling of similar activities have benefited from sampling techniques such as the time sampling strategy [10]. Vehicle-based sampling might not be suitable for a riding population with variable riding frequency and vehicle usage.

As pointed earlier, determining a sampling frame for the study also impose a potential challenge. Unlike cars or motorcycles, quad bikes are not under strict mandatory registration by Abu Dhabi Police or the Road and Transport Authority (RTA) in Dubai. Without compulsory registration, the number of quad bikes actively in use in the region is unknown. In addition, vehicle enumeration by quad biker’s rental agencies cannot be used as a sampling frame because riders hire these bikes for short periods only and/or in sites located away from home. Also, most quad bikes in the UAE are family-owned, mostly used on pool basis, i.e. with multiple riders to the same vehicle. Consequently, the use of quad bike registry in the UAE, a practice followed regularly for nation-wide vehicle surveys [11], would be impossible.

Identifying the number of active riders in the UAE was also very challenging in the absence of mandatory quad bike driver licensing rules. Unlike car drivers or motorcycle riders, getting a list of quad bike riders from any UAE province, based on the numbers of registered graduated drivers is not possible. As a consequence, no list of licensed riders exist, which could be used as a sampling frame, similar to vehicle users [12, 13]. As a result, we concluded that a sampling frame based on registered riders’ population was not possible due to the lack of mandatory vehicle registration and quad bike licensing.

Bias

Since this survey aims to describe riding behaviour, a biased response, including social desirability bias, is anticipated. Social desirability bias can make responses favouring safe riding habits [14–16]. The UAE has various regulations to ensure safe driving practices. Some regions have implemented safety laws like mandatory helmet use or banning the use of quad bikes on public roads [17]. We presume that many riders are aware of the rules and might ignore them in the absence of active enforcement. Thus, survey sampling strategies like telephone and online surveys in such an environment might run the risk of social desirability bias. This bias was reported in online surveys on safety behaviour and helmet use, where researchers have hinted at over-reporting helmet use [18–21]. To reduce this bias, we decided to observe the riders in their regular riding environment and to record their riding practices like helmet use, during the survey. This makes field-based observation and direct survey an essential feature of our survey sampling strategy.

In the absence of a rider or a vehicle-based sampling frame, we will use a location-based sampling frame with a list of all known riding sites in the UAE. With a growing urban population and riding areas close to the city region, we opted for location sampling methods similar to Kalton’s survey of homeless people at food shelters [22]. Kalton defines "location sampling" as sampling individuals visiting a specific location. It has two crucial sampling units, namely visitor and visits. This sampling strategy can produce a probability sample of visits [22]. Here, the visitor or quad bike driver, riding in the selected desert area, forms the unit of analysis. Location-based sampling has been widely used among nomadic populations and also for surveying wild birds around water sources [23]. In this study, "location" is not a fuelling station or home but is defined as a large desert area with a history of high quad bikes injury incidence.

Previous research on desert recreational activities have adopted various sampling strategies like satellite imagery and geospatial mapping [24, 25] or location-based visitor surveys in smaller parks [26, 27]. Location-based surveys in smaller parks might over-represent tourists and miss out riders in unorganized desert areas. Instead of focusing on a few riding parks, we want to cover larger unorganized riding spaces. Since quad bikes riders are known to be highly dispersed and transient in each location, we will follow the random geographic clustering method adopted by Himelein with nomadic pastoralists [28].

Desert-based travel for a dispersed rider population would entail many travel days for the sample collection. Moreover, travelling on the desert would require hiring a desert-worthy vehicle with an experienced driver. To reduce the cost of travel, we will limit data collection to the weekends during the winter riding season.

In addition, we will follow the line-transect method to reduce the cost of location sampling [29]. This method preserves some amount of randomness in identifying the dispersed population of quad bikes riders. The approach will systematically comb large desert areas for observation to undertake interviews with active quad bike riders. The line-transect sampling method or line-intercept sampling is usually employed to estimate the density or abundance of tree species, wildlife and marine fauna over a large area [25, 30]. It entails several lines of fixed length laid out in the region of interest and records the perpendicular distance of each animal from the line. Though the method helps estimating the number of sampling units within a large area, we could not use it to enumerate the number of quad bike riders owing to our limited budget for the desert travel. However, we will adopt the method to systematically comb vast desert areas for quad bike riders.

To conclude, we designed a new sampling strategy to observe quad bike riding practices for a highly seasonal, transient, dispersed quad biker population in the UAE.

## 2. Objective of the study

1. To describe driving habits and practices among quad bike drivers in the UAE, including all demographic subgroups and of all nationalities.
2. To estimate risky driving practices among quad bike drivers in the UAE.
3. To assess the knowledge levels of drivers about the safety measures and regulations related to driving quad bikes in the UAE.
4. To test the association between risk factors and outcome [Injury, risky driving].

In this article, we aim to present our innovative approach to creating a survey instrument based on a systematic review. Furthermore, we intend to introduce a novel sampling strategy that we have developed specifically for conducting this survey in the UAE.

### 2.1 Primary endpoint

#### 2.1.1 Injury: Percentage of riders getting injured that required hospital/emergency care

The survey aims to identify risky riding practices that result in injury. For this reason, we included self-reported injury as the primary endpoint to describe outcomes. Though endpoints are typically used for cohort and intervention studies, we will use them in this study to describe our riding population.

#### 2.1.2 Risky driving practices

Percentage of riders riding without a helmet.
Percentage of riders riding on paved roads.
Percentage of riders riding at night.
Percentage of riders carrying passengers.

A prior systematic review identified key risky riding practices such as riding without a helmet, riding on paved roads, riding at night and riding with passengers [31]. These practices were found associated with higher odds of crashes, injury and even death. Therefore, we included these modifiable risk behaviours as our primary endpoint for risky riding practices in the country. These endpoints are also chosen to inform policymakers on the possible intervention strategies in the region.

### 2.2 Secondary endpoint

Percentage of riders aware of safety laws and regulations relating to quad bike riding in the UAE.

Awareness of laws and regulations regarding safe driving habits was known to influence riding behaviour. Testing law awareness is an indispensable part of the survey, as it will inform policymakers on the awareness gaps. This in turn might influence the content of behaviour-change messaging. It will also help to identify the target population for this messaging.

## 3. Materials and methods

The study design is a cross-sectional observational study targeting active quad bike drivers in the UAE. The study will follow the STROBE checklist for observation studies [32] during its planning and reporting stages.

### 3.1 Operational definition

Quad bikes are four-wheeled all-terrain vehicles used for desert recreational sports in the UAE. The vehicles include electrical quad bikes and those driven by fossil fuel. The vehicle has several types, ranging from kid’s quad bikes, with an engine capacity of less than 90cc, to those with engine capacity greater than 500 cc, commonly classified as sports’ vehicles. Those with engine capacity of 150 to 500 cc are generally classified as newbie quad bikes. It is worthy noting that these classifications are based on a quad bike resource site [33]. Though these vehicles are also used for farming purposes, farmers are not included in the study.

As stated earlier, the injury outcomes which will be reported in this survey are self-reported quad bike injury incidents that needed medical care. These injuries may vary from skin lacerations requiring medical help only to fractures and traumatic brain injuries with more than two days of ICU or hospital stay. The questions identifying the severity of injury are worded around the number of days spent in the hospital.

Risky riding practices are practices proven elsewhere to increase the risk of quad bike crashes or increase injury severity. They include carrying passengers, riding on public roads, night riding and racing. Wearing personal protective equipment like helmets and goggles is included in this list.

### 3.2 Target group and sample size

The target group are active quad bike riders who ride for recreational purposes in the UAE. They include riders of both gender, demographic age groups and residency status. Only active riders will be included in this survey, while passengers or guardians will not be interviewed. Riding habits and vehicle descriptions will be observed and recorded before undertaking direct interviews with riders in desert locations.

Sample size estimation is based on a recent study in Ireland. The study showed helmet use in 16% of its riders population [34]. Keeping this as a rough indicator of risky riding behaviour, we set our expected prevalence rate at 0.12. The confidence interval was set at 95%, and the margin of error at 3%. The resulting sample size for this cross-sectional survey was calculated at 451 [n = Z^2^ P[1-P]/d^2^].

### 3.3 Sampling strategy

The survey will follow a three-staged, location-based sampling, using the line-transect method. A significant proportion of the riders’ population is expected to come from tourists and expatriates. This transient population could not be captured from any census data, so we aborted the proportionate sampling strategy to recruit a representative sample. To achieve that effectively we decided to recruit our sample from the three most populous Emirates of the UAE, namely, Abu Dhabi emirate (40%), Dubai (40%), and Sharjah (20%). The population of these emirates together represents 82% of the UAE population. To achieve a representative sample from the three emirates the study will use the same proportions (40%, 40% and 20%) to recruit the sampling units—desert locations, in each Emirate [Table 2 in S1 Appendix]. The following stage is to determine the sampling units, which consists of the target desert locations. These locations are areas in the desert with high incidence of quad bike injuries, identified from Ambulance records. These desert locations together, constitute the sampling frame for the study. Developing the sampling frame further is participatory with input from police, ambulance staff, gas station employees, survey participants and medical student volunteers. We will avoid over-representing frequent riders by making only one trip per location.

**Fig 1:**
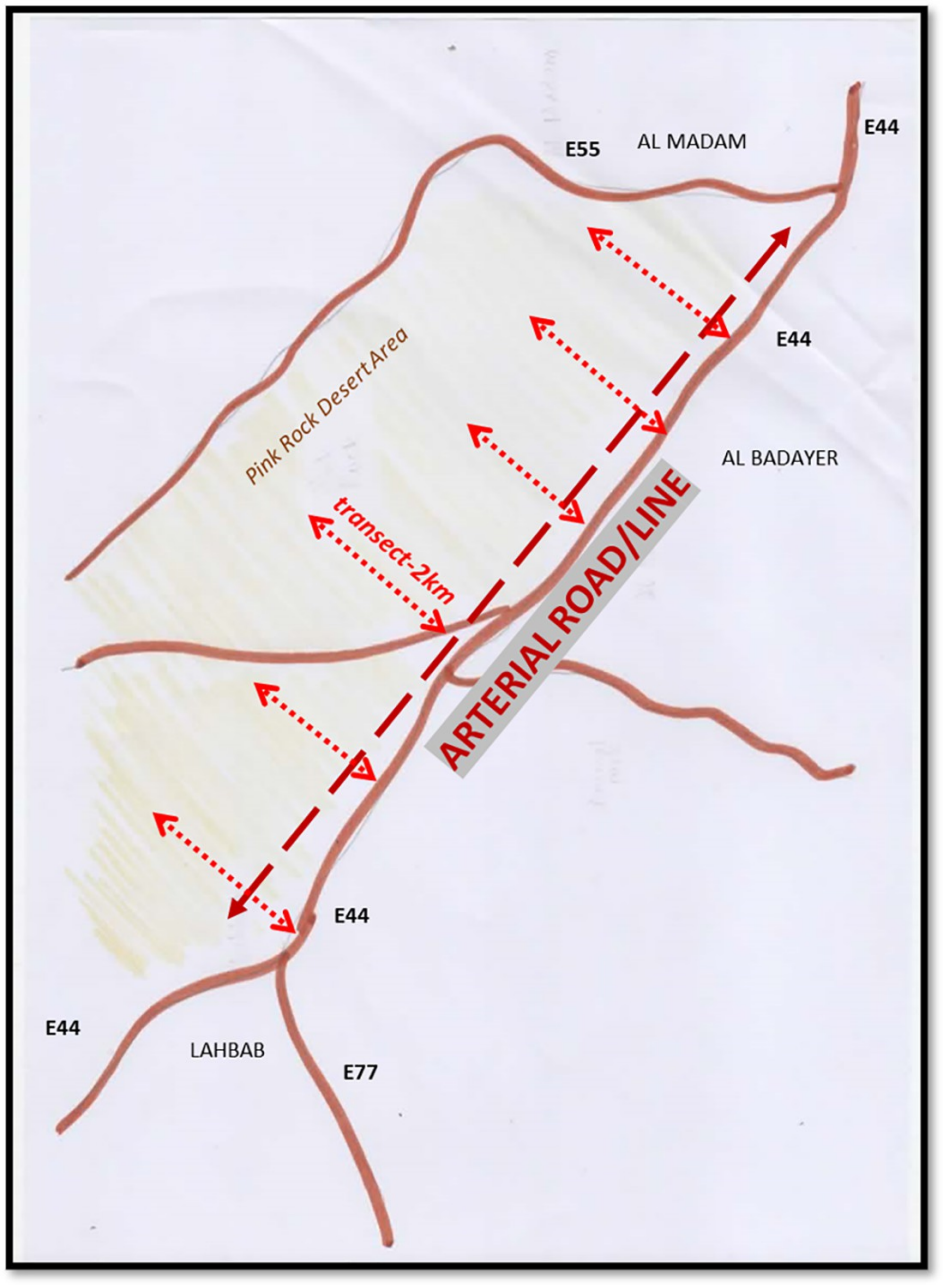
Line transect method: Riding 2 km into the desert from an arterial road.

To operationalize the line-transect method, we will first identify the arterial roads leading to the injury-prone desert areas of the sampling frame using Google Maps. A representative map is shown in [Fig 1], showing arterial roads that riders use to access the desert riding areas. The "lines" in this sampling method are the paved access roads to the selected desert "locations" [Fig 1].

**Fig 2:**
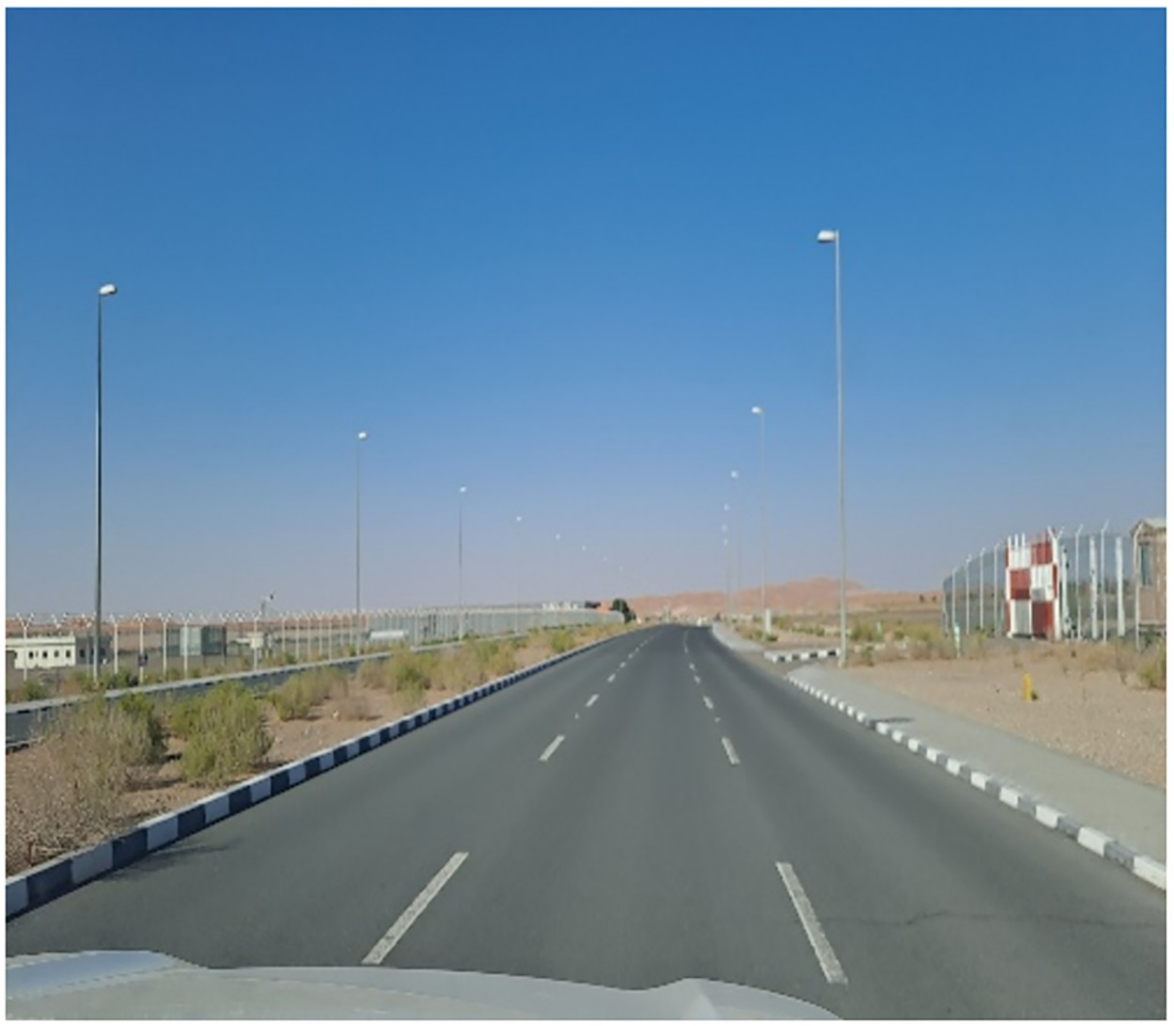
Arterial road [line] in area known for desert sport.

Each road has popular entry points adopted by local riders, easily identified by the track paths marked on the sand. We will select random stretches of 2 km paths for longer roads. Access to these locations usually stretches less than 2 kilometres [Fig 2].

**Fig 3:**
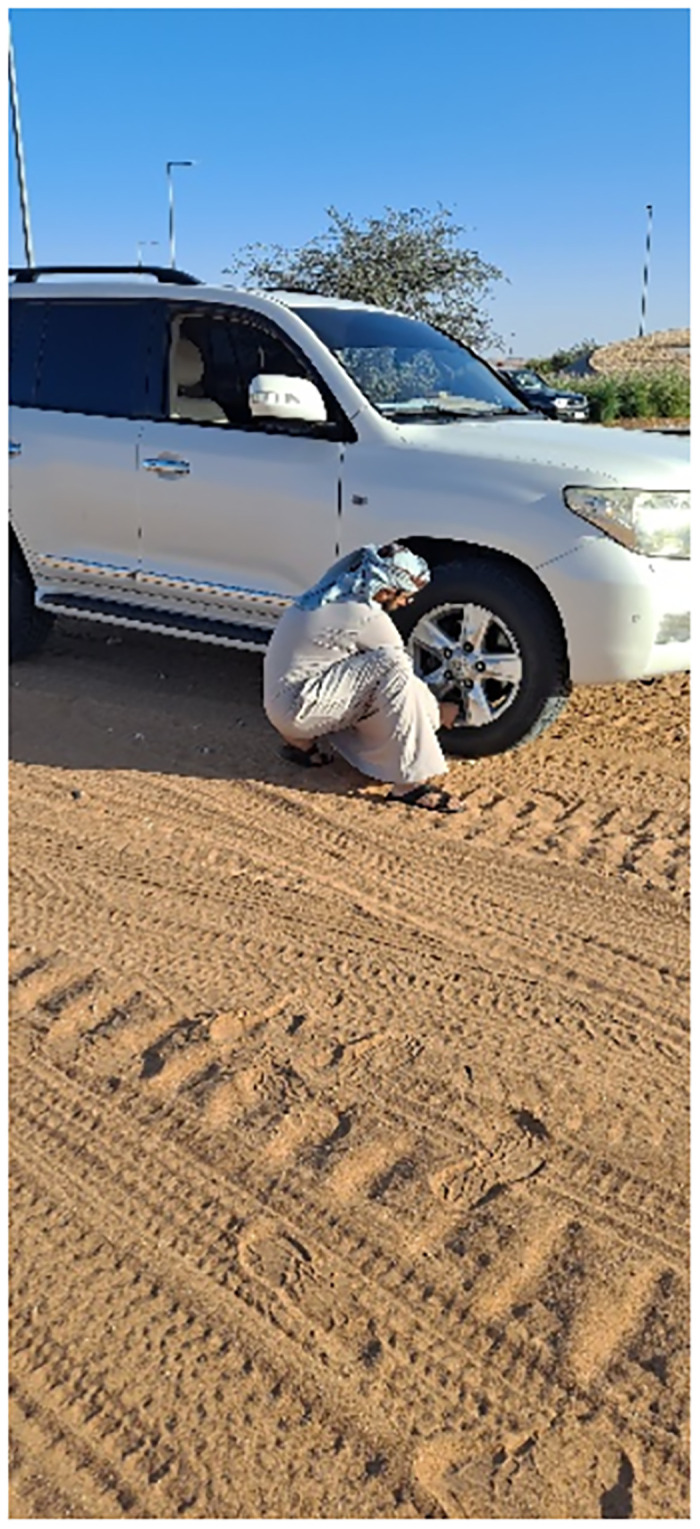
Entering desert at transect-reducing tyre pressure for desert ride.

The "transect" will be the trail followed by quad bike riders entering the desert from the arterial road [Fig 3].

**Fig 4:**
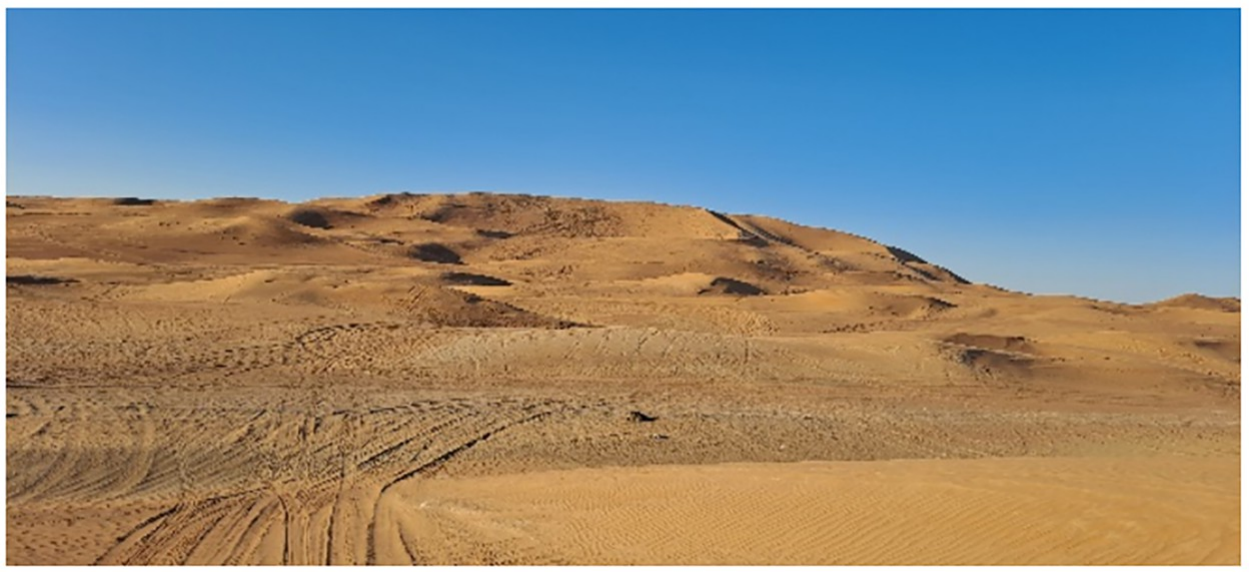
Following curved tracks [transect] around dunes to identify riders.

We will travel 2 to 5 kilometres into the dunes from each arterial road and followed a non-linear "transect" path [Fig 4]. Unlike the traditional transect path, the desert-transect route is not expected to be a straight line. Instead, the terrain would make us trace the contours of the dunes [Fig 4]. All riders in the visual region will be approached for the survey. Their driving behaviour will be observed before the survey and noted in the survey form.

### 3.4 Data collection strategy

Undergraduate student volunteers and a PhD student will administer the survey interviews and make field observations. The data collection team will consist of the researcher, biker volunteer, and vehicle driver. The surveys will be conducted during the evening hours of weekends or according to the time allocated by the tour operators.

All active riders in the sampled area will be approached for an interview. Guardians will be contacted before interviewing minors. The riders who refuse to be interviewed would be noted. Unsupervised children will not be approached. All participants will be briefed about the study in English or Arabic and will be interviewed after taking verbal consent. Written consent will be avoided following COVID-19 hygiene measures restricting fomite contact. Materials like pens, tablets or survey forms will not be shared with the participants to avoid direct contact. Most interviews will be documented in handheld tablets using Qualtrics Data Entry app [Qualtrics, Provo, UT]. Remote desert regions with poor connectivity, especially in the base of the dune or sand bowls, might necessitate offline data entry. Qualtrics can accommodate up to 20 offline interviews per device. Multiple tablets will be used when more than 20 participants aggregate in one location. Booklets with printed interviews would be used when multiple researchers are involved. Responses from printed interviews will be entered on the same day in the evening, together with uploading the app-based interviews to the cloud database. The printed and app-based interview questionnaires are identical, with visual prompts and questions in English and Arabic.

### 3.5 Data management plan

Survey responses collected in Qualtrics data platform will be downloaded from time to time by the first author. Only two researchers will have access to the database. The data will be stored in a hard disk drive with the date and the serial number of the survey response documented. The survey responses will be without identifiers like name or exact nationality. The cleaned data will be shared with the statistics expert. Validation and analysis will be shared and accessed by the authors. The survey data will not be shared with any third party and will not be used for any other study without an additional ethics approval.

### 3.6 Stakeholder support, approval, ethical clearance

The study was approved for research by the UAEU Research Ethics Sub-Committee for Social Sciences [No: ERS_2021_7342] and the Ethics Committee, Medical Accreditation and Research Department of Dubai Corporation for Ambulance Services, Dubai. Data collectors will be trained in the subject area, technical terminology, observation errors, observing ethics in survey studies, interviewing pediatric subjects and COVID hygiene regulations. Minors under 16 will be approached after taking the the guardian’s or parent’s verbal consent.

### 3.7 Survey tool

#### 3.7.1. Survey item source: Base tool and systematic review

The initial tool was based on the UNSW Quad bike Workplace Safety Survey tool [35]. This tool provided the template for all demographic and vehicle-based information. However, we note that our target population is not involved in farming activities. Since the UNSW tool does not delve into the risk factors needed for intervention design we modified the tool with survey items representing the risk factors for quad bikes injuries we derived from an earlier systematic review [31]. As mentioned earlier, our systematic review was focused on identifying the risk factors associated with quad bike crashes, injury or death among quad bike riders in all terrains. It comprised research articles from all over the world, all demographic and occupational groups. A list of all risk factors associated with quad bike crashes was identified of which only modifiable risk factors were considered for this survey [S1 File]. These risk factors were grouped into key survey domains such as inherent personal factors, vehicle factors, attitude and awareness. Since a direct observation is possible in the proposed field survey, sub-sections are grouped, based on observable driving habits [rider characteristics, vehicle characteristics and driving behavior] and self-reported information [driving history, injury history and awareness, emergency preparedness, and influencers].

#### 3.7.2 Transforming systematic review results to survey questions

Each risk factor derived from a systematic review is considered to form a survey item. These items are defined with an indication of how they influence health outcomes. These definitions are crucial in weeding out items that are expected not affect or represent the crash outcomes. Likewise, we eliminated those risk factors that are not measurable. Protective factors are duly noted on the direction of influence towards the crash. This may directly influence the numerical values allocated to answer options, especially for items identified as ordinal variables, with higher score given to options with higher risk for injury [S1 File].

The well-defined survey items will be classified as continuous, categorical, binary, and open-ended string variables. The possible outcomes for binary and categorical variables will be identified, listed and coded. The order of some categorical variables and their values will be based on their association with the crash outcomes [S1 File]. Some categorical variables, especially those related to self-reported injury outcomes, will be converted to ordinal variables. Continuous variables will be defined with the unit of measurement. Finally, open-ended questions will be excluded and used in a subsequent qualitative study.

The questions related to risk behaviour will be grouped for composite scoring criteria. The questions for very young subjects will not be rephrased since such questions would be administered on their parents/guardians. The tool was developed in English and later translated into Arabic by a student researcher. Thus, the survey items are defined, selected, characterized and grouped to create the questionnaire.

#### 3.7.3 Design and validation

The questionnaire was initially developed in Microsoft forms and further refined in Qualtrics [Qualtrics, Provo, UT] with inbuilt validation rules and travel paths. Furthermore, picture hints were added to a few response options to avoid misclassification. The questions were tested for face validity among English speakers and laypersons not involved in injury research or desert recreational sports. This tool was further validated for content from survey design and injury research experts. They were quizzed on the ease of understanding, flow and response fatigue.

Qualtrics questionnaire had both the English and Arabic version [S2 and S3 Appendices]. The questions were initially framed in English. They were translated into Arabic and later modified to accommodate regional dialects. Since Arabic speakers from the Middle East and North African countries use different dialects, we checked the Arabic version for errors and usage. Arab nationals from North Africa, the Levant and the Emirates tested the questionnaire for cultural appropriateness and clarity. The Arabic questionnaire was back-translated to English to check for consistency in meaning.

Midway through the data collection, we will use IBM SPSS Statistics Version: 28.0.0.0 [IBM Corp,UT] to perform Multiple Correspondence Analysis (MCA) or Factor Analysis of Mixed Data (FAMD). This process is intended to aid in validating the questionnaire for construct validity.

#### 3.7.4 Piloting and modification

The tool was piloted among 19 English and Arabic-speaking riders. The results were analyzed using the planned analysis protocol and presented in dummy tables. Several questions about risk perception had to be removed, as the responses were unclear in English and Arabic. These nuanced questions were removed from the survey and delegated to a subsequent qualitative study. Many participant parents did not consent to take measurements [anthropometric measurements] on their children, despite following COVID protocol and social distancing. Therefore, we altered the measuring instrument using a laser-guided goniometer and laser-guided tape. Since many participants did not consent to contact-less measurement, we dropped the survey item. The survey questions were reduced to 31 survey items in the final version [compared to 50 in the pilot] [S2 Appendix].

### 3.8 Survey items

The final survey questionnaire has seven major domains with 31 survey items. Injury history [6 items] and riding habits [8 items] are identified as outcome domains, while the other five domains will contribute to predictor variables in the risk model. Injury history covers injury experience, crash mechanism, severity, and treatment outcomes [S3 Appendix]. Riding habits captures risky riding behaviours such as carrying passengers, riding at night, using public roads and protective gear. Adhering to the survey’s aim of identifying and describing risky riding practices, practices like night-time riding, riding on paved roads, and carrying passengers will be treated both as outcomes for risky practices and also as predictors for injury. The five domains that contribute to predictors associated with injury or risky driving practices are sociodemographic characteristics [3 items], riding history [3 items], vehicle characteristics and ownership [3 items], other thrill-seeking behaviour [4 items], and awareness of regulation [4 items]. Riding history captures the age of initiation and training. Vehicle characteristics include brand, engine capacity and ownership. Thrill-seeking behaviour is captured by questions on other adventure sports’ experiences, over speeding, and racing. Similarly, participants’ awareness of various laws and regulations regarding quad bike riding, like helmet use, the ban on public roads, the minimum age limit for adult vehicles etc., will be recorded.

### 3.9 Analysis plan

**Fig 5:**
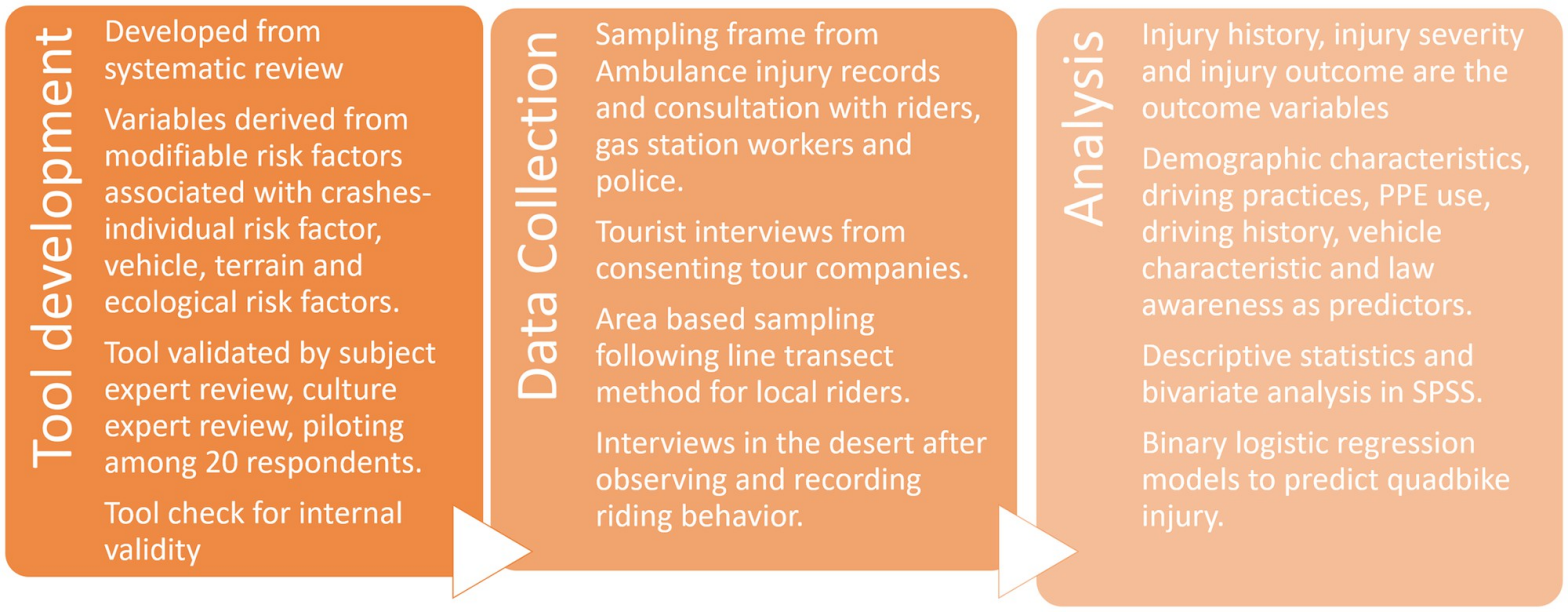
Study protocol.

We developed a detailed analysis plan accounting for each survey item. Each survey item was mapped to the analytical method and the results-dummy table. Each quad bike rider interviewed will form the unit of analysis. We will use Likert data to create outcome scores from various categorical variables. Our statistical analysis plan includes descriptive statistical methods and frequency tabulation. It would include inference analysis to test for association using ANOVA, Chi-square test, univariate and multivariate logistic regression. In addition, we planned for risk models with predictors for each outcome variable representing risky behaviour and injury. Since the survey tries to assess varied groups of risk factors like vehicle, training and initiation, riding preferences and risk-taking behavior. Latent class analysis will identify hidden constructs or risk groups. We will also employ cluster analysis [Two Step Cluster Analysis] to identify risk clusters. We will use Bayesian Information Criterion [BIC] and log-likelihood to test the validity of the measure. The study protocol has been summarized in Fig 5.

### 3.10 Dissemination

The results from the study will be shared with the police, Dubai Corporation for Ambulance Services (DCAS), injury prevention team at the Department of Health in Abu Dhabi, Ambulance and Emergency Preparedness sector in the UAE. Furthermore, abstracts of the results will be presented at various conferences, prioritizing local conferences for advocacy. That is in addition to publishing the methods and results in peer -reviewed journals.

## 4. Discussion

### 4.1 Strength

#### 4.1.1 Filling evidence gap

This study will explore the region’s much-neglected area of desert sports injuries. The study is the first of its kind in the UAE, with the objective of informing policymakers on injury prevention measures for quad bikes. Keeping intervention design as the main study goal, the study covers a wide range of modifiable risk factors associated with quad bike use in a desert environment. On the downside, this comprehensive approach may compromise the analytical power of the tool. However, it will hopefully capture evidence that would influence quad bike safety interventions.

#### 4.1.2 Direct observation and direct survey

The main strength of this survey is the credibility of the evidence. Only active quad bikers will be included, and the investigators will observe their riding behaviours. Observing a rider’s behaviour directly from the field is believed to be more credible than relying on self-reported evidence. We believe that direct observation could reduce the over-reporting of safe practices and under-reporting of risky practices that go against the law. This will further reduce the social desirability bias, as the researchers suspect that the riders may be aware of the safety laws regarding helmet use and public road use. The direct observation mentioned in the survey is limited to riding habits like wearing helmets, carrying passengers. Other details like injury experience and habits like nighttime riding or racing are self-reported, thus vulnerable to social desirability bias.

#### 4.1.3 Sampling strategy

This study will attempt to use a novel sampling strategy for a highly mobile and widely dispersed population of desert bikers. It is a location-based sampling method inspired by wild animal census [22, 23, 29]. The line-transect method is expected to work well for these desert sport vehicles as riders need to use a few arterial roads to reach their preferred desert riding destination. However, the sample may still be biased towards urban riders who use the streets to access the desert. It might under-represent rural riders who ride close to their homes/farms.

#### 4.1.4 Systematic tool development

Without a validated tool to meet the study requirement, the team had to develop a novel tool. This process involved a rigorous systematic review of all risk factors followed by a sequential conversion of this evidence to a list of measurable risk factors. The study followed a systematic and reproducible process of deriving the questions, identifying and coding answer options supported by a detailed analysis plan for each variable identified from the review. The tool, however, has to be reduced in length to accommodate the short attention span of riders in their desert environment. This tool development process could be replicated for evidence creation of other neglected public health issues.

#### 4.1.5 Offline data collection in remote desert locations

This survey adapts data collection methods for direct observation in the quad bike desert riding sites. It will maintain a uniform reporting format with offline tablet-based forms for poor network connectivity and paper entry options for an unanticipated large number of respondents, coupled with the traditional online forms in Qualtrics software [Qualtrics, Provo, UT, USA]. Qualtrics also accommodates graphical support to the answer options to reduce data entry errors during direct observation.

### 4.2 Limitations

Without any prior research on desert recreational sports injuries in the country, we had to take a calculated guess for sample size estimation and sampling strategy. Since most quad bikes in the country are unregistered, we have no reliable estimate of the number of active riders or quad bikes in use in the UAE. Vehicle enumeration might further under-represent the riders using rented or borrowed vehicles. This uncertainty is compounded by the varying tourist population who opt for desert riding sports. Without a rough estimate of the number of quad bike riders at risk of injury, we used hospital injury records, which might be the tip of the iceberg in quad bike injuries. Moreover, trauma hospital records in the country are fragmented among Emirates. Hence a nationwide estimate of injured riders is not possible to date.

Budgetary constraints restrict the days spent in direct desert-based observation and survey. The high cost of renting a desert all-terrain vehicle with a competent driver to navigate the trails in the line-transect sampling will consume a considerable amount of resources. Similarly, dispersed participants might compromise efficiency with fewer participants interviewed per trip. Low tourist flow due to possible travel ban might further compound this inefficiency.

The location-based sampling strategy might also contribute to response bias and selection bias. The age and nationality of the riders who refused to participate in the survey by riding away might not get documented. Non-responders, especially tourists and those with communication barriers might add to the selection bias.

## Supporting information

S1 Appendix Comparing various sampling strategies for sampling mobile population. (PDF)

S2 Appendix Quad bike survey questionnaire in Arabic. (PDF)

S3 Appendix Quad bike survey questionnaire in English. (PDF)

S1 File Step-by-step description of developing survey tool from systematic review results. (PDF)

S1 Checklist STROBE statement—Checklist of items that should be included in reports of observational studies. (PDF)

## References

[1] Vanlaar W, McAteer H, Brown S, Crain J, McFaull S, Hing MM. Injuries related to off-road vehicles in Canada. Accid Anal Prev 2015; 75:264–71. doi: 10.1016/j.aap.2014.12.006 25528439

[2] Bohl S. All- Terrain Vehicle Related Injuries and Hospitalization: An Examination of the Influence of Age and Substance Abuse [Doctoral Dissertation]. Minnesota: Walden University; 2010.

[3] Menon P, El-Sadig M, AB Khan M, Östlundh L, El-Deyarbi M, Al-Rifai RH et al. Risk factors associated with quad bike crashes: a protocol for systematic review of observational studies. BMJ Open 2021; 11[4]:e044456. doi: 10.1136/bmjopen-2020-044456 33820787 PMC8030468

[4] Consunji R, Malik S, El-Menyar A, Mollazehi M, Al-Thani H, Peralta R. Pediatric road traffic injuries in Qatar: Evidence for a developmental stage approach to road safety. Qatar Med J 2020; 2020[1]:3–8. doi: 10.5339/qmj.2020.3 32166071 PMC7052426

[5] Miller KW, Wilder LB, Stillman FA, Becker DM. The feasibility of a street-intercept survey method in an African-American community. Am J Public Health 1997; 87[4]:655–8. doi: 10.2105/ajph.87.4.655 9146448 PMC1380849

[6] Hader S. Sampling in Practice: GESIS Survey Guidelines. Mannheim, German: GESIS–Leibniz; December 2016.

[7] Wang Z, Neitzel RL, Xue X, Zheng W, Jiang G. Awareness, riding behaviors, and legislative attitudes toward electric bikes among two types of road users: An investigation in Tianjin, a municipality in China. Traffic Inj Prev 2019; 20[1]:72–8. doi: 10.1080/15389588.2018.1511898 30763127

[8] Wild H, Glowacki L, Maples S, Mejía-Guevara I, Krystosik A, Bonds MH et al. Making Pastoralists Count: Geospatial Methods for the Health Surveillance of Nomadic Populations. Am J Trop Med Hyg 2019; 101[3]:661–9. doi: 10.4269/ajtmh.18-1009 31436151 PMC6726942

[9] Morrison C, Lee JP, Gruenewald PJ, Marzell M. A Critical Assessment of Bias in Survey Studies Using Location-Based Sampling to Recruit Patrons in Bars. Subst Use Misuse 2015; 50[11]:1427–36. doi: 10.3109/10826084.2015.1018540 26574657 PMC5062950

[10] Mann J, Have TT, Plunkett JW, Meisels SJ. Time Sampling: A Methodological Critique. Child Development 1991; 62[2]:227–41. 2055121

[11] Khan M, Komanduri A, Pacheco K, Ayvalik C, Proussaloglou K, Brogan JJ et al. Findings from the California Vehicle Inventory and Use Survey. Transportation Research Record 2019; 2673[11]:349–60.

[12] Lynch CF, Logsden-Sackett N, Edwards SL, Cantor KP. The Driver’s License List as a Population-Based Sampling Frame in Iowa. Am J Public Health 1994; 84[4]:469–72. doi: 10.2105/ajph.84.3.469 8129069 PMC1614811

[13] Adimora AA, Schoenbach VJ, Martinson FE, Stancil TR, Donaldson KH. Driver’s License and Voter Registration Lists as Population-Based Sampling Frames for Rural African Americans. Annals of Epidemiology 2001; 11[6]:385–8. doi: 10.1016/s1047-2797(01)00230-7 11454497

[14] Trógolo M, Ledesma RD, Medrano LA. Validity and Reliability of the Attitudes toward Traffic Safety Scale in Argentina. Span. J. Psychol. 2019; 22. 10.1017/sjp.2019.54 31787124

[15] af Wåhlberg AE. Social desirability effects in driver behavior inventories. J Safety Res 2010; 41[2]:99–106. doi: 10.1016/j.jsr.2010.02.005 20497795

[16] Yılmaz Ş, Arslan B, Öztürk İ, Özkan Ö, Özkan T, Lajunen T. Driver social desirability scale: A Turkish adaptation and examination in the driving context. Transportation Research Part F: Traffic Psychology and Behaviour 2022; 84:53–64.

[17] The Use and Licensing of Recreational Motorbikes/Quad Bikes in the Emirate of Dubai: Regulation No. [4] of 2008; 2008 [cited 2021 Apr 3]. URL: https://www.rta.ae/wps/wcm/connect/rta/e3cf61df-e59c-4ff7-baf4-755467f9dbc5/Using_and_licensing_the_recreational_motorcycles_in_the_Emirate_of_Dubai.pdf?MOD=AJPERES&CACHEID=e3cf61df-e59c-4ff7-baf4-755467f9dbc5.

[18] Irwin A, Mihulkova J, Berkeley S, Tone L-R. ‘No-one else wears one:’ Exploring farmer attitudes towards All-Terrain Vehicle helmets using the COM-B model. J Safety Res 2022; 81:123–33. doi: 10.1016/j.jsr.2022.02.004 35589283

[19] Ledesma RD, Shinar D, Valero-Mora PM, Haworth N, Ferraro OE, Morandi A et al. Psychosocial factors associated with helmet use by adult cyclists. Transportation Research Part F: Traffic Psychology and Behaviour 2019; 65:376–88.

[20] Ruedl G, Abart M, Ledochowski L, Burtscher M, Kopp M. Self reported risk taking and risk compensation in skiers and snowboarders are associated with sensation seeking. Accid Anal Prev 2012; 48:292–6. doi: 10.1016/j.aap.2012.01.031 22664693

[21] Teschke K, Brubacher JR, Friedman SM, Cripton PA, Harris MA, Reynolds CCO et al. Personal and trip characteristics associated with safety equipment use by injured adult bicyclists: a cross-sectional study. BMC Public Health 2012; 12:765. doi: 10.1186/1471-2458-12-765 22966752 PMC3490930

[22] Kalton, Graham, editors. Practical Methods for Sampling Rare and Mobile Populations; 2001.

[23] Rivera-Milán FF, Collazo JA, Stahala C, Moore WJ, Davis A, Herring G et al. Estimation of Density and Population Size and Recommendations for Monitoring Trends of Bahama Parrots on Great Abaco and Great Inagua. Wildlife Society Bulletin 2005; 33[3]:823–34.

[24] Alrawaf TI, Abubakar IR, Alshabibi NM, Al-Matar KM, Dano UL, Elhadi EMA et al. The distribution of ecotourism activities and potential consequences for the Saudi desert ecosystem. Journal of Arid Environments 2023; 213:104950.

[25] Dewidar K, Thomas J, Bayoumi S. Detecting the environmental impact of off-road vehicles on Rawdat Al Shams in central Saudi Arabia by remote sensing. Environ Monit Assess 2016; 188[7]:396. doi: 10.1007/s10661-016-5400-6 27270484

[26] Kroeger T, Manalo P. Economic Benefits Provided by Natural Lands: Case Study of California’s Mojave Desert. Defenders of Wildlife; July 2007. URL: http://www.defenders.org/publications/mojaveeconomics.

[27] Wilkins EJ, Urioste-Stone S de. Place attachment, recreational activities, and travel intent under changing climate conditions. Journal of Sustainable Tourism 2018; 26[5]:798–811.

[28] Himelein K, Eckman S, Murray S. Sampling Nomads: A New Technique for Remote, Hard-to-Reach, and Mobile Populations. Journal of Official Statistics 2014; 30[2]:191–213.

[29] Glennie R, Buckland ST, Thomas L. The effect of animal movement on line transect estimates of abundance. PLoS One 2015; 10[3]:e0121333. doi: 10.1371/journal.pone.0121333 25799206 PMC4370374

[30] Jang W, Loh JM. Density estimation for grouped data with application to line transect sampling. Ann. Appl. Stat. 2010; 4[2].

[31] Menon P, El-Deyarbi M, Khan MA, Al-Rifai RH, Grivna M, Östlundh L et al. Risk factors associated with quadbike crashes: a systematic review. World J Emerg Surg 2022; 17[1]:27. doi: 10.1186/s13017-022-00430-2 35619139 PMC9137103

[32] The Equator Network. STROBE Statement—Checklist of items that should be included in reports of cross-sectional studies. URL: https://www.equator-network.org/reporting-guidelines/strobe/. 10.1007/s00038-007-0239-9 18522360

[33] ATV Helper. URL: http://atvhelper.com.

[34] Adil M, Konstantinou C, Porter DJ, Dolan S. All-Terrain Vehicle[ATV] Injuries–An Institutional Review Over 6 Years. Ulster Medical Journal 2017; 86[2]:103–7. 29535481 PMC5845989

[35] Transport and Road Safety Research Centre. Quad Bike and OPD Workplace Safety Survey Report: Results and Conclusion: SafeWork NSW. Sydney, Australia: University of New South Wales; 2017 May 31.

