# Supplementary material for: Collecting behavioral evidence from a highly mobile and seasonal population: A protocol for a survey on quad bike injuries
Source: PLoS One. 2024 Mar 4;19(3):e0298059. doi: 10.1371/journal.pone.0298059 (PMC10911601; doi:10.1371/journal.pone.0298059)
Supplement: S1 Appendix — (PDF) [file pone.0298059.s001.pdf]

Table 1: Various Sampling Strategies and Their Feasibility For Quadbike Survey

Sampling survey strategy	Reason for not selecting/selecting	Reference
**Telephone survey, Online survey**	Cannot observe participant vehicle fit, riding behaviour. Less validated observations	[1]Hader
**Using telephone book sampling frame; Gabler Hader design**	Will miss out non resident and children drivers. Anticipate low response rate.	[1]Hader
**Random Walk Method**	Quad bike sites are not uniformly distributed. Random selection not possible in desert. Quad biking sites may keep changing.	[1]Hader
**Arbeitsgemeinschaft Deutscher Markt- und Sozialforschungsinstitute (ADM) 3 stage cluster sampling**	Better representation. Getting ethics clearance from areas in norther emirates is a challenge. All riders in the cluster is good if the clusters are homogenous. But you have organized rentals and unorganized and spontaneous riding sites. How do you cater to this difference? Chance of missing out a demographic group.	[1]Hader; Selecting target municipalities[urban and semi urban] randomly, followed by selecting riding sites in that location randomly . Third stage is selecting all riders in that site on the specified interview date and time period
**2 stage sampling-population register sample**	No sampling frame for systematic sampling at second stage. Not restricted to any age demographic(6-15 years), ownership(have owners and renters). Risk of oversampling from organized quad biking sites.	[1]Hader; Primary sampling unit-municipalities. Second sampling stage-systematic random sample.
**Quota sampling**		
**Line transect method**	Though not applicable for rental and resorts, this method would be ideal to sample bikers who select varying[and spontaneous] location on the desert along a road. The police and ambulance have identified certain roads/stretches that were points for pick up. This is ideal for clusters of drivers who drive along the sand dune, that might be or might not be visible from the transect/road.	[2,3]Used in sampling mobile animals in wild areas. Useful for participants with no fixed location. The idea is to travel along the identified road. At various random points[based on tyre marks or parked cars] the researcher will travel along a transect perpendicular path for 1-2 kms into the desert. If he locates bikers, he will stop to interview, observe and enumerate the riders. If he did not find drivers, he will

		return back to the main road after two kms.
**Street intercept survey**	This is in addition to sampling registered establishment. Instead of street intercept survey, we identify biking roads, make transects and interview all those who we meet along the transect.<br>It best captures eligible population in a well defined geographical area. This best captures the mobile desert bikers. But it follows cluster sampling method and might have issues with variance in distribution. Also all drivers in the intercept are sampled, might end up in the same family, and might share vehicles between them[better representation of risk-vehicle fitness]	[3]Miller;Used to sample mobile urban populations in development projects in South Asia.<br>Shown to have high response rate and better representation of the mobile demographic group [men] when compared to household survey
**Stratified sampling**	Since we have two distinct riding environment, and each being a potential confounder to risk of crash, we can stratify our sample based on riding site. We stratify it into organized and unorganized driving environment. Organised could be driving rentals in built[tracks] or near desert tracks. Unorganised sites are those formed spontaneously when the group of riders or rider ventures out to the desert alone ontheir personal vehicle. At present we put it at the same proportion of sample for both strata. But there is a chance of over representation of organized driving sites. Unorganised samples are resource intensive[vehicle, driver, tracker/volunteer, researcher]	"Stratification is the process of segmenting our population across levels of some factor so as to minimize variability within those segments or strata."<br>https://www.itl.nist.gov/div898/handbook/ppc/section3/ppc332.htm<br><br>The focus here is not to ensure representativeness or accuracy in incidence rates. It is to ensure enough sample distribution to run different models for association.
**Random geographic cluster sample (RGCS)**	Not ideal for a highly mobile population. Circular clusters are difficult to reach from the arterial road and increase interviewing expense	[4]Himelein; Used for nomadic population sampling: In an RGCS design, the study area is stratified using data from Geographic Information Systems (GIS) sources. Within each stratum, points (latitude and

		longitude) are randomly selected, and then a circular cluster of a given radius is created around the point. All eligible respondents found within this cluster are selected for the survey.
**Water point survey**	Could be feasible by identifying bikes that come to petrol stations for re fueling. But may not see active driving and objective measurements. Driver and vehicle might be separate for children. It will also miss out tourists and rental.	[4]Himelein; Water point surveys are also common, but are biased as they exclude all livestock not found at a known watering point.
**Location sampling**	The biking site is the location, and sampling unit are the biker visit-not the biker. If a biker takes more than one visit during survey period, he will be excluded for his second visit. Here, primary sampling unit is a combination of location and time segment.	[5]Kalton; To sample individuals who visit specific locations such as libraries , museum, shopping centre, polling station or bars. Sampling occurs when a visitor enters or leaves the location. It produces probability sampling of visits if visits are taken as sampling unit.
**Area sampling**	This is ideal if the population of interest is evenly spread in an area. This is not the case for organized and unorganized biking. Hence not recommended.	Uneven spread rare population will have strata based on geographic location with disproportionate stratification.

Table 2: Using City Population to Decide Number Of Trips To High Injury Quadbiking Sites

CITY LIMITS FOR SURVEY	CITY POPULATION*	PERCENTAGE OF UAE POPULATION	NUMBER OF PARTICIPANTS IDENTIFIED	NUMBER OF TRIPS	NAME OF REGION WITH HIGH INJURY INCIDENCE OR KNOWN FOR DESERT SPORTS
**DUBAI**	1,137,347	0.3475	157	**16**	Al Awir, Lahbab, Khawaneej, leisaly, Rowaiya(50% of cases from Dubai Ambulance database)
**ABU DHABI**	603,492	0.184388	83	**8**	Al Khatim, Al Khazna, Al Wathba,Moreeb Dunes-Liwa (Referral from tour operators, riders, participants)
**SHARJAH**	543,733	0.16613	75	**7**	Badayer
**AL AIN**	408,733	0.124883	56	**6**	Al Nabbagh, Zakher, Ain Al Fayda, Strata Airport, Naggra
**TOTAL STUDY SITE REGION POPULATION**	2,693,305	0.822901			
**TOTAL SAMPLE SIZE**			**451**	**45**	

**[https://worldpopulationreview.com/countries/united-arab-emirates-population]*

References

1. Hader S. Sampling in Practice: GESIS Survey Guidelines. 2nd ed. Mannheim, German: GESIS – Leibniz; 2016.

2. Wang Z, Neitzel RL, Xue X, Zheng W, Jiang G. Awareness, riding behaviors, and legislative attitudes toward electric bikes among two types of road users: An investigation in Tianjin, a municipality in China. Traffic Inj Prev 2019;20(1):72-78. PMID:30763127

3. Miller KW, Wilder LB, Stillman FA, Becker DM. The feasibility of a street-intercept survey method in an African-American community. Am J Public Health 1997;87(4):655-658. PMID:9146448

4. Himelein K, Eckman S, Murray S. Sampling Nomads: A New Technique for Remote, Hard-to-Reach, and Mobile Populations. Journal of Official Statistics 2014;30(2):191-213. doi:10.2478/jos-2014-0013

5. Kalton, Graham, editors. Practical Methods for Sampling Rare and Mobile Populations; August 5-9; 2001.
