# Supplementary material for: Collecting behavioral evidence from a highly mobile and seasonal population: A protocol for a survey on quad bike injuries
Source: PLoS One. 2024 Mar 4;19(3):e0298059. doi: 10.1371/journal.pone.0298059 (PMC10911601; doi:10.1371/journal.pone.0298059)
Supplement: S2 Appendix — (PDF) [file pone.0298059.s002.pdf]

العربية

Consent, Biking history, Demographic

هذا استطلاع يجريه طلاب من جامعة الإمارات العربية المتحدة حول استخدام الدراجات الرباعية.
تحاول هذه الدراسة فهم تفضيلاتك في قيادة الدراجة الرباعية و تجربة القيادة.
نود أن نطرح عليك بعض الأسئلة التي لن تستغرق سوى 6 دقائق من وقتك.
نحن لا نسجل اسمك أو رقم الاتصال الخاص بك. ستكون ردودك جنبًا إلى جنب
مع ردود السائقين الآخرين مجهولة المصدر و لا يمكن لأي شخص تتبعها إليك.
ستكون جميع المعلومات التي تقدمها سرية ولن تتم مشاركتها مع أي شخص.
هذه المقابلة طوعية ويمكنك اختيار التوقف في أي وقت. لا تتردد في مشاركة
أي من مخاوفك أو شكوكك مع بريتا مينون[201990141@uaeu.ac.ae].
هل ترغب في مشاركة تجربتك في ركوب الدراجات الرباعية؟

- نعم ○
- لا ○

الرجاء تحديد الجنس (الردود مجهولة)

- أنثى ○
- ذكر ○

العمر

[ ]

الجنسية / الإقامة

- إماراتي ○
- خليجي ○
- وافد مقيم ○
- سائح ○

Yamaha	Can Am	STELS
○ Aeon	○ CF Moto	○ Suzuki
○ Kawasaki	○ KTM	○ أخرى
○ Polaris	○ Kymco	○ لا أذكر
○ Kayo		

حدد سعة المحرك

○ 50	○ 125	○ 250	○ 400	○ 570	○ 1000 or more
○ 70	○ 150	○ 270	○ 450	○ 700	○ محرك كهربائي
○ 90	○ 200	○ 350	○ 525	○ 800	○ لا أذكر
○ 110	○ 220				

حدد الملابس الواقية (حدد أقسامًا متعددة إذا لزم الأمر)

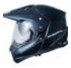
☐ خوذة

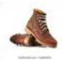
☐ أحذية طويلة

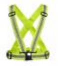
☐ سترة / عاكسة

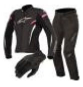
☐ ملابس ضيقة

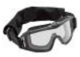
☐ نظارات واقية

يرجى وضع علامة على الخيار الأنسب. هل يمكنك إخبارنا عدد المرات التي تمارس فيها عادات الركوب التالية ؟ [إجابتك سر

	دائما	بعض الأحيان	أبدا
هل تمنح الآخرين توصيلة كركاب ؟	○	○	○

هل قدت أي من المركبات التالية في الصحراء؟ (يمكنك اختيار أكثر من مركبة)

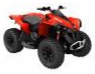
☐ دراجة رباعية

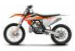
☐ Dirt bike

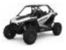
☐ Side by side/buggy

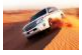
☐ 4X4 off road car

كم مرة تقود الدراجات الرباعية ؟ (الموسم: أكتوبر إلى أبريل)

- مرة أو مرتين فقط ○
- نادراً (أقل من 4 مرات في الموسم) ○
- مرة أسبوعياً خلال الموسم ○
- أكثر من 3 أيام في الأسبوع خلال الموسم ○
- يومياً أثناء الموسم أو الإجازة ○

في أي عمر بدأت قيادة الدراجات الرباعية لأول مرة؟

- ○ تحت 6 سنوات
- ○ 11-16 سنة
- ○ 6-10 سنوات
- ○ فوق 16 سنة

من علمك ركوب الدراجات الرباعية أو قدم لك المشورة ؟ (حدد أقسامًا متعددة إذا لزم الأمر)

- ☐ تعلم ذاتي
- ☐ معهد تدريبي
- ☐ أحد أفراد الأسرة الأكبر سنا
- ☐ وكالة تأجيردراجات
- ☐ الأقران / الأصدقاء
- ☐ أخرى

هل تمتلك هذه الدراجة الرباعية أو استعرتها من العائلة أو الأصدقاء أو استأجرتها في وكالة تأجيردراجات ؟ (تعتبرملكية العائلة إذا كانت مشتركة بين أفراد العائلة)

- ○ ملكية شخصية
- ○ وكالة تأجيردراجات
- ○ مستعارة من العائلة أو من صديق
- ○ أخرى

حدد الشركة المصنعة للدراجة الرباعية

○ ○ ○

	دائما	بعض الأحيان	أبدا
هل تقود على طريق ممهد ؟	○	○	○
هل ترتدي خوذة أثناء القيادة ؟	○	○	○
هل تقود في الظلام / بعد غروب الشمس ؟	○	○	○
هل تتسابق في أحداث رسمية أو غير رسمية بالدراجات الرباعية ؟	○	○	○

نوع الخوذة (حدد أقسامًا متعددة إذا لزم الأمر)

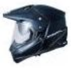
○ Full face

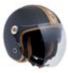
○ Half face

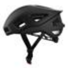
○ Cycling

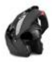
○ Modular

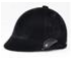
○ Equestrian

هل تزداد احتمالية ارتدائك للخوذة في الحالات التالية : (حدد أقسامًا متعددة إذا لزم الأمر)

- ☐ بحضور أحد الأبوين / ولي الأمر
- ☐ حدث رياضي / حدث سباق
- ☐ عند القيادة على أرض غير مألوفة
- ☐ بحضور جهات إنفاذ القانون (الشرطة)
- ☐ وقت النهارفقط، للوقاية من الشمس
- ☐ أخرى
- ☐ في وكالات تأجير الدراجات أو تأجير الساحات
- ☐ عندما بدأت القيادة لأول مرة، توقفت الآن

حدد الإمارة \ المنطقة التي تقود فيها دراجتك الرباعية (حدد أقسامًا متعددة إذا لزم الأمر)

- أبوظبي ☐
- دبي ☐
- راس الخيمة ☐
- الشارقة ☐
- أم القيوين ☐
- الفجيرة ☐
- عجمان ☐

Injury History

☐ احتجت إلى الذهاب للطوارئ أو زيارة العيادة الخارجية
☐ إعادة التأهيل، العلاج الطبيعي
☐ احتجت إلى البقاء في المستشفى\العناية المركزة لمدة يومين أو أقل
☐ أخرى ______

Self Reported Confidential

يرجى مشاركة عدد المرات التي تقوم فيها بما يلي. (إجابتك سرية ومجهولة المصدر ، ولا يمكن للباحث رؤية ردك)

	بانتظام	بعض الأحيان	مطلقا
التدخين (شيشة، سيجارة إلكترونية، سجائر، مدواخ)	○	○	○
استهلاك المشروبات الكحولية	○	○	○
قيادة السيارات والدراجات بسرعة عالية	○	○	○
رياضات المغامرة (القفز بالحبال، والطيران المظلي)	○	○	○

Emergency Preparedness

ما هو الرقم الذي ستتصل به اذا شاهدت حادثة دراجة رباعية خطيرة ؟

☐ لا أدري
☐ العائلة / صديق مقرب
☐ 997/998/999
☐ أخرى ______

Influencer

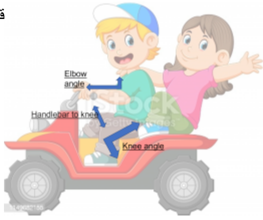

قياسات: من فضلك ، هل يمكنني أخذ القياسات أثناء جلوسك على الدراجة ؟

الطول (سم) ☐
الوزن(كجم) لك الحرية في التزام الصمت إذا كنت لا ترغب في كتابة وزنك ☐

الرجاء الإجابة إذا كانت القوانين أو العبارات التالية صحيحة في إمارتك التي تقود الدراجة فيها ؟

	لا أدري	نعم	لا
هل قيادة الدراجات الرباعية على الشارع غير قانوني ؟	○	○	○
هل يمكن للأطفال الذين تقل أعمارهم عن 16 عاما قيادة دراجات البالغين ؟	○	○	○
هل ارتداء الخوذ إلزامي، وعدم ارتداء الخوذة قد يؤدي إلى غرامات ؟	○	○	○

خلال السنة الماضية (الموسم\ الشتاء الماضي)، كم عدد المرات التي فقدت فيها التحكم بالدراجة حيث اضطررت الى القفز أو رميت من الدراجة بعد ميلانها أو إنقلابها ؟

○ لم يحدث
○ 1-3 مرات
○ أكثر من 3 مرات

في السنوات الخمس الماضية ، هل سبق لك أن تعرضت لحادث اصطدام على دراجة رباعية احتاجت إلى رعاية طبية؟

○ لا
○ نعم، أكثر من مرة
○ نعم، مرة واحدة
○ نعم ، على مركبات أخرى غير الدراجة الرباعية

في أي وقت من اليوم وقع الحادث ؟

○ وقت النهار
○ ليل
○ وقت مساء
○ لا أذكر

ما هو سبب الإصابة التي تعرضت لها ؟ (حدد أقسامًا متعددة إذا لزم الأمر)

☐ 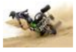 رميت من الدراجة
☐ اصطدمت مع مركبة أخرى أو سطح صلب
☐ 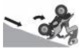 الدراجة انقلبت
☐ 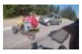 القيادة على الشارع
☐ 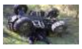 علقت تحت الدراجة
☐ أخرى ______

أي جزء من الجسم أصيب ؟ (حدد أقسامًا متعددة إذا لزم الأمر)

☐ الرأس، الرقبة، المخ، العمود الفقري، الأعصاب
☐ الرئتين، العيون، الوجه، الكبد، الأمعاء
☐ العضلات، العظام
☐ أخرى ______

ماذا كانت نتيجة تلك الاصابة ؟ (حدد أقسام متعددة إذا لزم الأمر)

☐ عالجت الإصابة بنفسي
☐ احتجت إلى البقاء في المستشفى\العناية المركزة لمدة 3 أيام أو أكثر

هل يمكنك تسمية أي قناة يوتيوب، أو حساب فيسبوك، أو شخصية مشهورة، أو حساب إنستغرام، أو تويت، تتابعه بانتظام لركوب الدراجات الرباعية ؟ (يمكنك كتابة أكثر من جواب واحد)

______

اسم الباحث

☐ Preetha
☐ Hazza
☐ Saleh
☐ Mansoor
☐ Marwan
☐ Abdullah
☐ Humaid
☐ Other ______

UAE Quad bike Study

بواسطة Qualtrics
