# Supplementary material for: Collecting behavioral evidence from a highly mobile and seasonal population: A protocol for a survey on quad bike injuries
Source: PLoS One. 2024 Mar 4;19(3):e0298059. doi: 10.1371/journal.pone.0298059 (PMC10911601; doi:10.1371/journal.pone.0298059)
Supplement: S3 Appendix — (PDF) [file pone.0298059.s003.pdf]

English

Consent, Biking history, Demographic

UAE Quad Bike Study
This is a survey conducted by students of UAE University on Quad Bike use. This study is trying to understand your ATV or quad bike driving preferences and driving experience. We would like to ask you a few questions which will only take 6 minutes of your time. We are not recording your name or contact number. Your responses along with those of other drivers will be anonymous and nobody can trace it back to you. All information that you give will be confidential and not be shared with anyone. This interview is voluntary and you can choose to stop at any time. Feel free to share any of your concerns or doubts with Preetha Menon [201990141@uaeu.ac.ae].

Would you like to share your quad bike riding experience?

- ○ Yes
- ○ No

Have you driven any of the following off-road vehicles in the desert?

- ☐ Quad bike/All terrain vehicle

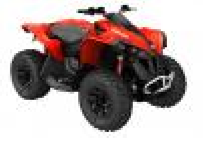

- ☐ Dirt bike

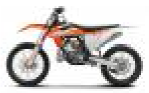

- ☐ Side by side/buggy

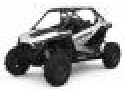

- ☐ 4X4 off road car

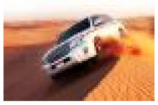

How frequently do you ride quad bikes [Season means October to April]?

- ○ Just once or twice

- Rarely [less than 4 times per season]
- Once every week during the season
- More than 3 times a week during the season
- Everyday during the season or during vacation

At what age did you first start driving quad bikes?

- Less than 6 years of age
- 6- 10 years
- 11-16 years
- >16 years

Who taught you to ride a quad bike? Or give you driving tips?

- Self-taught
- Older Family Member
- Peer/Friend
- Training Institute
- Rental Agency
- Netizen

Do you own the quad bike that you usually ride or do you borrow it from family, friend or rental agency[family ownership regarded if shared between family members] ?

- Owned
- Borrowed from family members
- Rental agency
- Borrowed from friends

Which quad bike do you usually ride?

- Yamaha
- Aeon
- Kawasaki
- Polaris
- Kayo
- Can Am
- CF Moto
- KTM
- Kymco
- STELS
- Suzuki
- Other
- Can't remember

Can you share the engine capacity [cc]of the quad bike that you usually ride?

- 50
- 125
- 250
- 400
- 570
-

					1000 or more
○ 70	○ 150	○ 270	○ 450	○ 700	○ Electric
○ 90	○ 200	○ 350	○ 525	○ 800	○ Don't remember
○ 110	○ 220				

Which of the following protective gear do you usually wear while riding quad bikes?

- ☐ Helmet 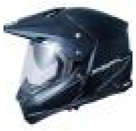
- ☐ Boots 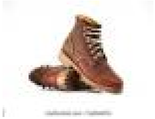
- ☐ Reflective clothing 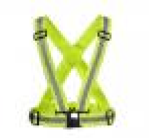
- ☐ Biking outfit 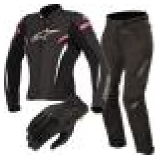
- ☐ Goggles 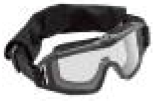

Can you tell how frequently you do the following ? Please tick on the most appropriate option.

	Always	Sometimes	Never
Do you give others a ride as passengers?	○	○	○
Do you ride on paved road?	○	○	○
Do you wear helmet while driving?	○	○	○

	Always	Sometimes	Never
Do you drive after sunset/in the dark?	○	○	○
Do you race on quad bikes-formal events or informal races?	○	○	○

Which type of helmet do you usually use?

- ☐ Full face 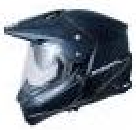
- ☐ Half face 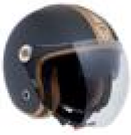
- ☐ Cycling 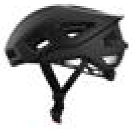
- ☐ Modular 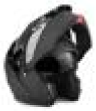
- ☐ Equestrian 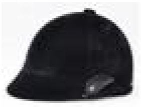

Are you more likely to wear a helmet when:

- ☐ In the presence of parent/guardian
- ☐ Sporting events/Race
- ☐ Only on unfamiliar terrain
- ☐ In the presence of law enforcement/police
- ☐ Depends on weather- as sun/wind protection
- ☐ When helmet is available
- ☐ Rental agencies or rental riding parks
- ☐ When I first started driving , now discontinued

Can you share some information about yourself [responses are anonymous]?

Your Gender

- ○ Female
- ○ Male

Can you share your age [in years]?

Nationality/Residence status

- ○ Emirati
- ○ GCC
- ○ Expatriate resident
- ○ Tourist

In which Emirate/Region do you usually drive your quad bike?

- ☐ Abu Dhabi
- ☐ Dubai
- ☐ Ras Al Kaimah
- ☐ Sharjah
- ☐ Umm Al Quwain
- ☐ Fujairah
- ☐ Ajman

Injury History

In the last one year; how many times have you lost control of your quad bike that made you jump off the quad bike, or be thrown off the bike, after it rolled over or tipped?

- ○ None
- ○ 1-3 times
- ○ >3 times

In the last 5 years, have you ever experienced a crash on quad bike that needed medical care?

- ○ No
- ○ Yes, once
- ○ Yes, more than once
- ○ Yes , on other vehicles other than quad bike

At what time did the crash occur

- ○ Day : Morning-Noon
- ○ Evening
- ○ Night
- ○ Don't remember

What was the cause of the most serious injury

- ☐ Was thrown off the quad bike 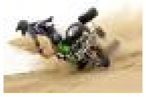
- ☐ The quad bike rolled over 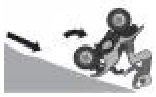
- ☐ I was pinned under the quad bike 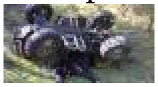
- ☐ Crashed with other vehicle or hard surface
- ☐ Crash on road 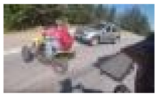
- ☐ Other ____________

What part of the body was injured[select multiple sections if needed]?

- ☐ Head, Neck, Brain, Spinal cord, Spine
- ☐ Skeletomuscular system
- ☐ Soft tissue-lungs, eye, face, liver, GI system
- ☐ Other ____________

What was the outcome of that injury [select multiple sections if needed]?

- ☐ Injuries treated by self
- ☐ Injuries needing Emergency care or outpatient visit
- ☐ Injury needed ICU/hospital admission for upto 2 days
- ☐ Injury needed ICU/hospital admission for more than 3 days
- ☐ Rehabilitation, physiotherapy
- ☐ Other ____________

Self Reported Confidential

Confidential: Please share how frequently you do the following. Your response is confidential and anonymous, the researcher cannot see your response.

	Regularly	Occasionally	Never
Smoking [sheesha, vape, cigarette, dhokha]	○	○	○

	Regularly	Occasionally	Never
Drink alcohol	○	○	○
Drive cars and bikes at high speed	○	○	○
Adventure sports [bungee jumping, paragliding, zipline]	○	○	○

Emergency Preparedness

Which number will you call when you witness a serious quad bike accident?

- ☐ Don't know
- ☐ 997/998/999
- ☐ Family, close friend
- ☐ Other ____________________

Influencer

Can you name any you-tuber, celebrity, famous personality, instagram account, twitter or facebook page that you regularly follow for quad biking?

____
