# Supplementary material for: Collecting behavioral evidence from a highly mobile and seasonal population: A protocol for a survey on quad bike injuries
Source: PLoS One. 2024 Mar 4;19(3):e0298059. doi: 10.1371/journal.pone.0298059 (PMC10911601; doi:10.1371/journal.pone.0298059)
Supplement: S1 File — (PDF) [file pone.0298059.s004.pdf]

STEP 1: Risk Factors Identified from Systematic Review

Concept Block	Risk Factor
Driver Characteristic	Age
	Gender
	Nationality
	Residency Status
	Occupation
	Years of residency in UAE or middle east
Vehicle Characteristic	Type
	Brand
	Fuel
	Engine Size
	Gear
	Seat to footrest length
	Presence of Vehicle Crush Protective Equipment
	Altered vehicle
	Ownership-Bought/rented/borrowed
	Vehicle permanence
Driver Vehicle Fit	Height
	Weight
	Arm Span
	Elbow bent angle
	Foot brake coverage
	Seat clearance -Distance from vehicle seat when standing
	Handle bar knee distance
Driving History	Driving history
	Frequency of driving history
	Age of driving intiatition
	First age of riding quadbike as passenger
	Who taught them to ride
	Underwent organised training for quadbikes
	Where did they go for organised driving lessons

Concept Block	Risk Factor
**Injury History**	History of past loss of control in the last month
	Loss of control occurrence time
	Loss of control with same vehicle
	Experienced a crash that needed medical attention
	Serious crash occurrence time
	If serious crash occurred, what happened then
	Having witnessed a serious crash requiring medical attention
	Knowing any aquaintance, who suffered a serious quad bike crash
**Driving Behaviour**	Carrying passenger
	Children, driving adult vehicle
	Driving on paved roads
	Wearing helmet
	Type of helmet
	Helmet chin strap
	Ownership helmet
	Enviroment inducing helmet use
	Seatbelt observation
	Fastening seatbelt
	Driving in dark
	Racing
	Wearing goggles, shoes
	Speed limit
	Speed limit if present
	Maximum speed of riding
**Awareness**	Awareness of Dubai Traffic law on quadbike safety-no driving under age of 16, no driving on paved roads, no carrying passengers
	Awareness-Age of permitted driving-16
	Awareness -Ban on driving on paved road and residential areas
	Awareness-Law for use of helmet
**Emergency preparedness**	Awareness of emergency contact in case of injury
	Do you carry a mobile phone with you at all times-or any one of your driving group
	Trained in first aid

Concept Block	Risk Factor
**Risk Perception**	Hazard awareness
	Injury perception
**Influencers**	Influencer in neighborhood
	Social media influencer
**For children-parent supervision**	adult supervision
**Risk taking habit**	Risk behavior-smoking, driving at high speed-other vehicle, adventure sports
	Regular Smoking
	Regular Alcohol Consumption
	Regularly driving fast cars/bikes
	Street racing
	Frequently doing other adventure sport-bungee jumping, gliding, mountain climbing, horse or camel race

STEP 2: Measurable Risk Factor Suited for Survey

Variables/Survey Items	Justification/Hypothesis
**Driver Characteristics**	
Age	Age relation with riding behaviour, vehicle, injury, driving history, awareness of law, risk perception, attitude
Gender	Gender relation with riding behaviour, vehicle, injury, driving history, awareness of law, risk perception, attitude
Nationality	Identify nationality and possible target language
Residency Status	To identify Emirati, expats ratio with visitors and tourists-focus of intervention, language of intervention
Occupation	To identify blue collared workers, sedentary workers, white collared worker and student composition
Years of residency in UAE or middle east	How long the residents have been related to knowledge of laws, emergency
**Vehicle Characteristic**	
Type	Predominant use of sports vehicle vs other and the age distribution for these use-do they follow the law? Wo
Brand	Dominant brand if they have option of adding protective equipment
Fuel	
Engine Size	Engine size and age mismatch, engine size and fall history
Gear	Use of helmet, goggles, boots, tight outfit
Seat to footrest length	Vehicle fit-for active riding
Presence of Vehicle Crush Protective Equipment	Risky behaviour, ownership relation
Altered vehicle	If it is altered, used for racing-risky behavior
Ownership-Bought/rented/borrowed	To see if there is freedom to fit protective equipments, and related to vehicle misfit, also rental agencies taking precautions
Vehicle permanence	If they are using the same vehicle for the last one month[window period for injury]- if not, vehicle misfit is not applicable
**Driver Vehicle Fit**	
Height	To check if age or hieght is a better fit-for riding safety. And if related to vehicle misfit- -to be used later in allocating correct vehicle during purchase from dealer or during renting
Weight	To check if weight is more correlated to injury than age when it comes to this vehicle--to be used later in allocating correct vehicle during purchase from dealer or during renting
Arm Span	If arm span is a better indicator of misfit than age-to be used later in allocating correct vehicle during purchase from dealer or during renting
Elbow bent angle	Measure of vehicle fit-related to injury or loss of control history

Variables/Survey Items	Justification/Hypothesis
Foot brake coverage	Measure of vehicle fit-related to injury or loss of control history
Seat clearance -Distance from vehicle seat when standing	Measure of vehicle fit-related to injury or loss of control history
Handle bar knee distance	For active riding
**Driving History**	
Frequency of driving	
Age of driving intiatition	Age of driving initiation could have some link to risk behaviour-policy implication on age of license and training
First age of riding quadbike as passenger	Those riding as passengers at younger age might get initated sooner than other-could lead to risky driving
Who taught them to ride	To identify influencers, and who should the target be for campaign
Underwent organised training for quadbikes	to know the percentage of riders who went through organised training, if it is more among those who are participating in events-identify correlation with injury-could be reverse causation though-this also identifies stakholders, influencers
Where did they go for organised driving lessons	Any specific training institute-for stakeholder study
**Injury History**	
History of past loss of control in the last month	A proxy measure of crash, or pre-injury. This outcome is tested for its association with various other risk factor. Recall period is one month. So we cannot do this study in the beginning of driving season.
Loss of control occurrence time	Night crash vulnerability
Loss of control with same vehicle	To make sure vehicle fit does not influence injury
Experienced a crash that needed medical attention	A proxy measure of serious injury. Recall period for this is long, when compared to the loss of control variable.
Serious crash occurrence time	Night crash
If serious crash occurred, what happened then	Experience after crash-if it influences riding behavior
Having witnessed a serious crash requiring medical attention	This variable is to see if witnessing these incidents has a positive influence on driving safety measures. A measure of self-efficacy.
Knowing any aquaintance, family or friend who suffered a serious quad bike crash resulting in hospitalisation or surgery	This variable is to see if witnessing these incidents has a positive influence on driving safety measures. A measure of self-efficacy. If the relationship is strong, then education material should include crash visuals or sensitize people to the consequences.

Variables/Survey Items	Justification/Hypothesis

Driving Behaviour (by direct observation)	
Carrying passenger	One of the risky driving practise. Check for association with other risk factors like age, gender, having ridden as a passenger, and awareness with law.
Children, driving adult vehicle	One of the risky driving practise. Check for association with other risk factors like age, gender, and awareness with law.
Driving on paved roads	One of the risky driving practise. Check for association with other risk factors like age, gender, and awareness with law.
Wearing helmet	Protective driving practise. This has to be corroborated with data collector observation.
Type of helmet	Safety guidelines adherence
Helmet chin strap	
Ownership helmet	Role of ownership on use
Enviroment inducing helmet use	Motivation for helmet use
Seatbelt observation	This can be observed. But cannot be classified as risky or protective. Some sources say that seatbelt hinders easy jumping off the vehicle,-protective strategy against crash injury
Fastening seatbelt	This can be observed. But cannot be classified as risky or protective. Some sources say that seatbelt hinders easy jumping off the vehicle,-protective strategy against crash injury
Driving in dark	One of the risky driving practise. Check for association with other risk factors like age, gender,ownership and awareness with law.
Racing	One of the risky driving practise. Check for association with other risk factors like age, gender,engine size, training and awareness with law.
Wearing goggles, shoes	Protective clothing direct observation
**Awareness**	
Awareness-Age of permitted driving-16	This is to assess knowledge of law. If less, intervention should be to increase awareness. Also to check self efficacy, if people are flouting the law despite being aware of it.
Awareness -Ban on driving on paved road and residential areas	
Awareness-Law for use of helmet	
**Emergency preparedness**	
Awareness of emergency contact in case of injury	To assess their knowledge of this vital life saving information. If low, then awareness program must include it.
Trained in first aid	To assess the gap. This skill is important for reducing morbidity and mortality. If it should be included in licensing/training module.

Variables/Survey Items	Justification/Hypothesis
**Risk Perception**	
Hazard awareness	To correlate hazard perception with risky driving behaviour
Injury perception	To correlate risk perception with risky driving behavior
Influencers	
Influencer in neighborhood	To identify people that they look up to. These influencer demographic could be used for delivering intervention message. They could be converted first for safe driving behavior.
**Risk Habit**	
Adult supervision	To assess the prevalence of unsupervised driving among minors[this being a group that is not legally entitled to drive quadbikes]
Risk behavior-smoking, driving at high speed-other vehicle, adventure sports	To check if risk driving behaviour is a part of risk taking personality-need to check for other risky behaviour practise like smoking, driving cars or bikes at high speed, racing on road.
Regular Smoking	
Regular Alcohol Consumption	
Regularly driving fast cars/bikes	
Street racing	
Adventure sport-bungee jumping, gliding, horse or camel race	

CODE LIST -VARIABLES WITH VARIABLE CODE [FOR ANALYSIS]

Variable code	Option Code
SD_Age	Numeric
SD_Gen	Male-1/Female-0
SD_Nat	Emirati-3/GCC-2/Expatriate Resident-1/Tourist-0
VC_cat	Sports Quad /Utility quad 4 wheels/ Utility Quad 6 Wheels/MiniQuad/Junior Quad
VC_br	Yamaha-0/Suzuki-1/Aeon-2/Can Am-3/Polaris-4/KTM-5/Kayo-6/Cant remember, Other-7
VC_eng	Numeric
VC_eng_cat	Youth ATV=0/Teen and new riders=1/Sport ATV=2
Ow_Ow	"Owned",0,"Borrowed from family members",1,"Borrowed from friend", 1 ,"Borrowed
Dh_V_qb	Yes-1/No-0
Dh_V_db	Yes-1/No-0
Dh_V_bg	Yes-1/No-0
Dh_V_4	Yes-1/No-0
Dh_time	Less than 1 year/1-2 year/2-3 year/3-5 year/>5 year
Dh_AnyRec	Yes-1/No-0
Dh_Other Rec	Yes-1/No-0
Dh_freq	Just once or twice-0/Rarely [less than 4 times per season]-1/Once every week during the season-2/ More than 3 times a week during the season-3/ Everyday during the season or during vacation-4
Dh_AgIni	"Less than 6 years of age", 3, "6-10 years", 2,"11-16 years",1,">16 years",0
Dh_Teach	"Self-taught",3,"Older Family Member",1,"Training Institute-0, "Peer/Friend",2,"Rental
Ih_Lc	"None",0,"1-3 times",1,">3times",2

Ih_Inj	"No"0,"Yes, once",1,"Yes, more than once",2,"Yes , on other vehicles other than quad bike",3
Ih_time	"Day: Morning - Noon", 0,"Evening", 1,"Night", 2,"Don't remember",3
Ih_mech	Was thrown out of the vehicle-1/Bike rolled over me-0/pinned under the vehicle-3/crashed with
Ih_mech_coll road	Crash on road or ride on road-1, Other crash mechanism-0
Ih_mech_collveh	Crash with other vehicles on the desert-1, Other crash mechanism-0
Ih_mech_rollover	Rollover-1, Other-0
Ih_mech_pinned	Pinned-1, Other -0
Ih_mech_ehect	Eject-1, Other-0
Ih_org_h	Yes-1/No-0
Ih_org_b	Yes-1/No-0
Ih_org_s	Yes-1/No-0
Ih_org_sk	Yes-1/No-0
Ih_org_o	Yes-1/No-0
Ih_org_o_desc	String
Ih_out_s	Yes-1/No-0
Ih_out_ op	Yes-1/No-0
Ih_out_ ip2	Yes-1/No-0
Ih_out_ ip3	Yes-1/No-0
Ih_out_ pt	Yes-1/No-0
Ih_out_o	Yes-1/No-0
Ih_out_o_desc	String
Ih_out	"Injuries treated by self",1,"Injuries needing Emergency care or outpatient visit",2,"Injury Injury needed ICU/hospital admission for more than 3 days,4,"No injuries",0

Db_Pass	Sometimes",1 ,"Rarely",1 ,"Never",0
Db_Road	Always-2/Sometimes-1/Rarely-1/Never-0
Db_Helm	Always-0/Sometimes-1/Rarely-1/Never-2
Db_Night	Always-2/Sometimes-1/Rarely-1/Never-0
Db_Race	Always-2/Sometimes-1/Rarely-1/Never-0
Db_AdVh	Always-2/Sometimes-1/Rarely-1/Never-0 "Full face",0,
Db_Htype	"Modular",0,"Half face", 1,"Equestrian", 1,"Cycling",1
Db_HStr	Yes-0/ No-1
Db_Reas_Helm_parent	Yes-1/No-0
Db_Reas_Helm_race	Yes-1/No-0
Db_Reas_Helm_ter	Yes-1/No-0
Db_Reas_Helm_pol	Yes-1/No-0
Db_Reas_Helm Wind	Yes-1/No-0
Db_Reas_Helm_av	Yes-1/No-0
Db_Reas_Helm_park	Yes-1/No-0
Db_Gear_h	Yes-0/No-1
Db_Gear_ b	Yes-0/No-1
Db_Gear_ v	Yes-0/No-1
Db_Gear_ o	Yes-0/No-1
Db_Gear_ g	Yes-1/No-0
Db_gear_all	Yes-1/No-0

Aw_Rd	"Don’t know",1,"Yes",0,"No",2
Aw_16	"Don’t know"1,"Yes",2,"No",0
Aw_H	"Don’t know",1,"Yes",0,"No",2<br>999-998/997-0/Family member-1/Don’t know-2/Other-0
Aw_Num	"Don't know",2,"Family, close friend",1,"997/998/999",0,"SOS",0
Aw_Fa	Yes-1/no-0
Rb_Sm	"Regularly",2,"Occasionally",1,"Never",0
Rb_Al	Regularly-2/occasionally-1/never-0
Rb_dr	Regularly-2/occasionally-1/never-0
Rb_Rc	Regularly-2/occasionally-1/never-0
Rb_As	Regularly-2/occasionally-1/never-0
