# Supplementary material for: Collecting behavioral evidence from a highly mobile and seasonal population: A protocol for a survey on quad bike injuries
Source: PLoS One. 2024 Mar 4;19(3):e0298059. doi: 10.1371/journal.pone.0298059 (PMC10911601; doi:10.1371/journal.pone.0298059)
Supplement: S1 Checklist — (PDF) [file pone.0298059.s005.pdf]

STROBE Statement—checklist of items that should be included in reports of observational studies

	Item No.	Recommendation	Page No.	Relevant text from manuscript
**Title and abstract**	1	(*a*) Indicate the study's design with a commonly used term in the title or the abstract	1	*…a Protocol for a Survey on Quad Bike Injuries*
		(*b*) Provide in the abstract an informative and balanced summary of what was done and what was found	1	*This is a protocol for study that aims to describe quad bike rider behaviour and assess the risk factors associated with injuries in the Middle East.*
**Introduction**				
Background/rationale	2	Explain the scientific background and rationale for the investigation being reported	2-5	*Evidence on quad bike crashes, their risk factors, societal burden and the most effective measures to control the problem in the UAE is minuscule. This research attempts to fill the gap in the existing literature on quadbikes injuries in the UAE.*
Objectives	3	State specific objectives, including any prespecified hypotheses	6	*1. To describe driving habits and practices among quad bike drivers in the UAE, including all demographic subgroups and of all nationalities.*<br>*2. To estimate risky driving practices among quad bike drivers in the UAE.*

				3. To assess the knowledge levels of drivers about the safety measures and regulations related to driving quad bikes in the UAE.<br>*4. To test the association between risk factors and outcome [Injury, risky driving].*
**Methods**				
Study design	4	Present key elements of study design early in the paper	6	*The study design is a cross-sectional observational study targeting active quad bike drivers in the UAE*
Setting	5	Describe the setting, locations, and relevant dates, including periods of recruitment, exposure, follow-up, and data collection	8	*Data collection strategy*
Participants	6	(*a*) *Cohort study*—Give the eligibility criteria, and the sources and methods of selection of participants. Describe methods of follow-up<br>*Case-control study*—Give the eligibility criteria, and the sources and methods of case ascertainment and control selection. Give the rationale for the choice of cases and controls<br>*Cross-sectional study*—Give the eligibility criteria, and the sources and methods of selection of participants	7,8	*The target group are active quad bike riders who ride for recreational purposes in the UAE. They include riders of all gender, demographic age groups and residency status. Only active riders would be included in this survey, while passengers or guardians would not be interviewed.*
		(*b*) *Cohort study*—For matched studies, give matching criteria and number of exposed and unexposed		Not applicable

		Case-control study—For matched studies, give matching criteria and the number of controls per case		
Variables	7	Clearly define all outcomes, exposures, predictors, potential confounders, and effect modifiers. Give diagnostic criteria, if applicable	15	*The final survey questionnaire has seven major domains with 31 survey items. Injury history (6 items) and riding habits (8 items) are identified as outcome domains, while the other five domains will contribute to predictor variables in the risk model….*
Data sources/ measurement	8*	For each variable of interest, give sources of data and details of methods of assessment (measurement). Describe comparability of assessment methods if there is more than one group	*11*	*Data Management Plan detailed.*
Bias	9	Describe any efforts to address potential sources of bias	4, 17,18,19	*To avoid this bias, we decided to observe the riders in their regular riding environment and to record their riding practices like helmet use, during the survey. This makes field-based observation and direct survey an essential feature of our survey sampling strategy.*
Study size	10	Explain how the study size was arrived at	9	

	Sample size estimation is based on a recent study in Ireland. This study showed helmet use in 16% of riders…

Continued on next page

Quantitative variables	11	Explain how quantitative variables were handled in the analyses. If applicable, describe which groupings were chosen and why	16	Analysis plan detailed.
Statistical methods	12	(*a*) Describe all statistical methods, including those used to control for confounding	16	Detailed analysis plan in page.
		(*b*) Describe any methods used to examine subgroups and interactions		Depends on the data. Will address them later. This is a protocol paper.
		(*c*) Explain how missing data were addressed		Protocol paper. N/A
		(*d*) *Cohort study*—If applicable, explain how loss to follow-up was addressed<br>*Case-control study*—If applicable, explain how matching of cases and controls was addressed<br>*Cross-sectional study*—If applicable, describe analytical methods taking account of sampling strategy		Protocol paper. N/A
		(*e*) Describe any sensitivity analyses		Protocol paper. N/A
**Results**				
Participants	13*	(a) Report numbers of individuals at each stage of study—eg numbers potentially eligible, examined for eligibility, confirmed eligible, included in the study, completing follow-up, and analysed		Protocol paper. N/A
		(b) Give reasons for non-participation at each stage		Protocol paper. N/A
		(c) Consider use of a flow diagram		Protocol paper. N/A
Descriptive data	14*	(a) Give characteristics of study participants (eg demographic, clinical, social) and information on exposures and potential confounders		Protocol paper. N/A
		(b) Indicate number of participants with missing data for each variable of interest		Protocol paper. N/A
		(c) *Cohort study*—Summarise follow-up time (eg, average and total amount)		Protocol paper. N/A
Outcome data	15*	*Cohort study*—Report numbers of outcome events or summary measures over time		Protocol paper. N/A
		Case-control study—Report numbers in each exposure category, or summary measures of exposure		Protocol paper. N/A
		Cross-sectional study—Report numbers of outcome events or summary measures		Protocol paper. N/A
Main results	16	(*a*) Give unadjusted estimates and, if applicable, confounder-adjusted estimates and their precision (eg, 95% confidence interval). Make clear which confounders were adjusted for and why they were included		Protocol paper. N/A
		(*b*) Report category boundaries when continuous variables were categorized		Protocol paper. N/A
		(*c*) If relevant, consider translating estimates of relative risk into absolute risk for a meaningful time period		Protocol paper. N/A

Continued on next page

Other analyses	17	Report other analyses done—eg analyses of subgroups and interactions, and sensitivity analyses	Protocol paper. N/A
**Discussion**			
Key results	18	Summarise key results with reference to study objectives	Protocol paper. N/A
Limitations	19	Discuss limitations of the study, taking into account sources of potential bias or imprecision. Discuss both direction and magnitude of any potential bias	19 Limitations detailed within Discussion section.
Interpretation	20	Give a cautious overall interpretation of results considering objectives, limitations, multiplicity of analyses, results from similar studies, and other relevant evidence	Protocol paper. N/A
Generalisability	21	Discuss the generalisability (external validity) of the study results	19 Mentioned in the discussion section. Limited relevance in protocol paper.
**Other information**			
Funding	22	Give the source of funding and the role of the funders for the present study and, if applicable, for the original study on which the present article is based	This section has been added in the application. Removed from the manuscript.

*Give information separately for cases and controls in case-control studies and, if applicable, for exposed and unexposed groups in cohort and cross-sectional studies.

Note: An Explanation and Elaboration article discusses each checklist item and gives methodological background and published examples of transparent reporting. The STROBE checklist is best used in conjunction with this article (freely available on the Web sites of PLoS Medicine at http://www.plosmedicine.org/, Annals of Internal Medicine at http://www.annals.org/, and Epidemiology at http://www.epidem.com/). Information on the STROBE Initiative is available at www.strobe-statement.org.
